# Rhodoglobus galactosidasius sp. nov., a psychrophilic Actinomycetota producing a cold-active β-galactosidase isolated from Cape Hallett, Antarctica

**DOI:** 10.1099/ijsem.0.007263

**Published:** 2026-09-16

**Authors:** Andrea Cattaneo, Luigi Leone, Ida Romano, Marianna Pannico, Barbara Nicolaus, Paola Di Donato, Annarita Poli, Ilaria Finore

**Affiliations:** 1 National Research Council of Italy (CNR), Institute of Biomolecular Chemistry (ICB), via Campi Flegrei 34, Pozzuoli, 80078 Napoli (NA), Italy; 2 Ca’ Foscari University of Venice, Department of Environmental Sciences, Informatics and Statistics (DAIS), via Torino 155, 30172 Mestre (VE), Italy; 3 National Research Council of Italy (CNR), Institute of Polymers, Composites and Biomaterials (IPCB), via Campi Flegrei 34, Pozzuoli, 80078 Napoli (NA), Italy; 4 Parthenope University of Naples, Department of Science and Technology (DiST), Centro Direzionale - Isola C4, 80143 Napoli (NA), Italy

**Keywords:** Antarctica, Cape Hallett, β-galactosidase, novel species, psychrophile, *Rhodoglobus*

## Abstract

A novel psychrophilic member of the phylum *Actinomycetota*, designated strain CPHL_1^T^, was isolated from the shoreline of Cape Hallett, Antarctica. Cells were Gram-positive, aerobic, non-motile, non-spore-forming rods (0.7–1.0 µm in length) with orange to yellow pigmentation. Optimal growth occurred at 15 °C, pH 8.0, with NaCl tolerance up to 3% (w/v). Strain CPHL_1^T^ exhibited cytosolic cold-active β-galactosidase activity, with an optimum at 15 °C and pH 7.0 and formed non-adherent biofilms. Phylogenetic analysis based on 16S rRNA gene sequence placed the strain within the genus *Rhodoglobus*, showing high similarity to *Rhodoglobus vestalii* LV3^T^ (99.4%) and *Rhodoglobus aureus* CMS 81y^T^ (97.6%). Chemotaxonomic characteristics, including the predominance of anteiso-C_15 : 0_, anteiso-C_17 : 0_ and iso-C_16 : 0_ fatty acids, the presence of MK-11, MK-10 and MK-12 as the major respiratory quinones, and genomic G+C content of 60%, further supported its assignment to the genus *Rhodoglobus*. Whole-genome comparisons revealed clear taxonomic distinction, with strain CPHL_1^T^ sharing 88.9% average nucleotide identity (ANI) and 38.3% digital DNA–DNA hybridization (dDDH) values with *R. aureus*, and 81.7% ANI and 25.1% dDDH with * R. vestalii*, confirming its status as a novel species. Phylogenetic analysis revealed that the β-galactosidases from these *Rhodoglobus* strains form a well-supported clade with high sequence similarity. Based on these data, a novel species is proposed: *Rhodoglobus galactosidasius* sp. nov. (type strain CPHL_1^T^ =ITEM 19986^T^ =KCTC 59592^T^). Genomic analyses also highlighted its biosynthetic potential for specialized metabolites, consistent with the recognized metabolic diversity of the genus.

## Introduction

The genus *Rhodoglobus* was originally proposed by Sheridan *et al*. [1] and is currently assigned to the class *Actinomycetes* and the family *Microbacteriaceae* [2, 3]. At the time of writing, only two species with validly published names are recognized, namely *Rhodoglobus aureus* CMS 81y^T^ [4, 5] and *Rhodoglobus vestalii* LV3^T^ [1], both isolated from cyanobacterial mat samples collected from Antarctic ponds on the McMurdo Ice Shelf.

Some genera within the family *Microbacteriaceae* were recently reclassified because of blurred boundaries between new species [6]. Moreover, the type strain of *Leifsonia rubra* is no longer available for study and was, therefore, not considered [5, 7].

Bacterial diversity in Antarctica remains underexplored by the scientific community. It has been estimated that more than half of autotrophic microbial species have not yet been described, with comprehensive surveys largely restricted to specific terrestrial regions, such as the McMurdo Dry Valleys [8]. To isolate microorganisms belonging to rarely recovered or recently defined genera, cultivation strategies employing oligotrophic media and prolonged incubation have been used to favour the growth of slow-growing bacteria [9]. Phylogenetically diverse microbial communities inhabit extreme environments, including cryospheric ecosystems (temperature <5 °C), where they are shaped by strong selective pressures and environmental stressors [10].

In this study, the chemotaxonomic and phylogenetic characteristics of the novel psychrophilic strain CPHL_1^T^ are reported. This strain produces a cold-active β-galactosidase, and it was isolated from Antarctic coastal sediments collected at Cape Hallett, within Antarctic Specially Protected Area no. 106. Based on its phenotypic features and distinct phylogenetic placement, *Rhodoglobus galactosidasius* sp. nov. is proposed for strain CPHL_1^T^.

## Methods

### Sampling, bacterial isolation and culture conditions

Water and sediment samples were collected in January 2018 from the shoreline of Cape Hallett (Antarctic Specially Protected Area no. 106, Antarctica; 72° 20.533′ S, 170° 09.860′ E), during the XXXIII Italian Antarctic Expedition. The sampling site was characterized by low temperature (−0.7 °C) and pH 6.5. Samples were stored at −20 °C until analysis, then slowly thawed and gently homogenized. Serial dilutions of the water fraction were prepared, and aliquots of 50 µl were spread onto modified R2A agar (yeast extract 0.2 g l^−1^, peptone 0.2, casamino acids 0.2, sodium pyruvate 0.3, MgSO_4_ · 7H_2_O 0.05, agar 18.0). Plates were incubated at 15 °C for 25 days.

A yellow-orange-pigmented colony, designated strain CPHL_1^T^, was isolated by repeated streaking on R2A agar until purity was achieved [11]. Strain CPHL_1^T^ represented the fastest-growing microorganism on modified R2A agar and was the only isolate recovered at the highest dilutions. Sub-culturing was performed in tryptic soy broth (TSB; pancreatic digest of casein peptone 17.0 g l^−1^, papain digest of soya peptone 3.0, NaCl 5.0, glucose 2.5, K_2_HPO_4_ 2.5) for 96 h at 15 °C. Long-term preservation was achieved as freeze-dried cells in 10% (w/v) skimmed milk at 25 °C in the CE-ICB Extremophiles Collection, and in CIBAN-MNA Italian Antarctic Bacterial Culture Collection-Italian National Antarctic Museum.

### orphological, physiological and biochemical analyses

Cell and colony morphology was examined using phase-contrast microscopy (Nikon Eclipse E400), and a stereomicroscope (Leica M8), respectively. Scanning electron microscopy (SEM) was performed following a protocol adapted from Romano *et al*. [12]. Cells were grown in TSB medium (15 °C for 96 h), harvested by gentle centrifugation (1,000 x g, 15 min, 4 °C) and washed with 0.02 M phosphate buffer pH 7.0 (Na_2_HPO_4_⋅7H_2_O 3.10 g l^−1^, NaH_2_PO_4_ ⋅H_2_O 1.16). Fixation was performed in 2.5% (v/v) glutaraldehyde in the same buffer for 20 h at room temperature, followed by washing. Samples were dehydrated in a graded ethanol series, air-dried at room temperature and coated with 10 nm Au/Pb using a sputter coater. Observations were performed with a FEI Quanta 200 FEG SEM at 5 kV (EDT).

Gram reactions, oxidase and catalase activities, hydrolysis of starch, cellulose and gelatin, motility, spore formation and nitrite/nitrate reduction were assessed according to standard procedures [11–13]. Pigment extraction was performed as previously described [4]: freeze-dried cells were extracted with methanol (1 g dry weight:20 ml), and UV-visible spectra were recorded between λ 200–1,000 nm using a Genesys 150 UV-Vis spectrophotometer (Thermo Fisher Scientific). Growth under aerobic conditions was tested in liquid and solid (agar 1.8%, w/v) R2A, TSB and Luria-Bertani (LB; tryptone 10.0 g l^−1^, yeast extract 5.0 and NaCl 10.0) media at 15 °C.

Biofilm formation was evaluated in static cultures (modified R2A medium supplemented with sucrose 1%, w/v) at 15 °C for 12 days, using polystyrene Petri dishes as support. Optimal growth parameters were determined in TSB by varying temperature (4–35 °C), salinity (NaCl 0.5–10.0%) and initial pH (3–10) while maintaining the previously determined optimal parameters. Optical density (OD) at λ 600 nm and pH (PHM210 pH metre; Radiometer) were measured after 96 h incubation at 120 r.p.m. All experiments were performed in triplicate, and mean values are reported.

Carbon source utilization and chemical sensitivity were assessed using the GEN III MicroPlate (Biolog), incubated at 20 °C for 8 days following the manufacturer’s instructions using a culture grown on TSB agar and inoculating fluid A (Biolog). Assays were performed in triplicate and results were manually interpreted. Selected carbon utilization profiles were validated in modified R2A supplemented with 1% (w/v) individual sugars (d-glucose, d-fructose, d-maltose, d-mannose, sucrose), and growth was quantified by OD measurements after 96 h (15 °C, 120 r.p.m.).

### Chemotaxonomic characteristics and *in silico* chemotaxonomic analyses

For chemotaxonomic analyses, strain CPHL_1^T^ was grown in TSB at 15 °C for 48 h with shaking. Cells were harvested at the stationary phase and freeze-dried.

Quinones were extracted from freeze-dried cells with *n*-hexane, purified by TLC on silica gel plates (0.25 mm; 60 F_254_, Merck) and eluted with *n*-hexane/ethyl acetate (96 : 4, v/v). Purified UV-visible bands were analysed by LC/MS using a reverse-phase RP-18 Lichrospher column eluted with *n*-hexane/ethyl acetate (99 : 1, v/v) at a flow rate of 1.0 ml min^−1^, and identified by electrospray ionization mass spectrometry and ^1^H NMR spectrometry [14]. NMR spectra were recorded at the Institute of Biomolecular Chemistry of CNR (Pozzuoli, Italy) using a Bruker DPX-300 spectrometer operating at 300 MHz with a dual probe. The residual pellet, after *n*-hexane extraction, was further extracted with CHCl_3_/CH_3_OH/H_2_O (65 : 25 : 4, v/v/v) for polar lipids recovery. Polar lipids were analysed by TLC using the same solvent system. Total lipids were detected by spraying with 0.1% (w/v) Ce(SO_4_)_2_ in 1 M H_2_SO_4_ or with 3% (w/v) methanolic molybdophosphoric acid, followed by heating at 100 °C for 5 min. Phospholipids, aminolipids and glycolipids were detected using Dittmer-Lester reagent, ninhydrin and α-naphthol, respectively [15]. Fatty acid methyl esters were obtained from total lipids as previously reported [13]. Cell wall monosaccharides were analysed from hydrolysates prepared as previously reported [16]. Aliquots of the hydrolysates were applied onto silica gel plates (0.25 mm; 60 F_254_; Merck) together with monosaccharide standards (arabinose, galactose, mannose, xylose, rhamnose, ribose and glucose) for identification. Chromatographic separation was performed using ethyl acetate/acetic acid/2-propanol/formic acid/water (25 : 10 : 5 : 1 : 15, v/v/v/v/v) as mobile phase in a saturated chamber. Plates were developed for 1 h at room temperature. Sugars were visualized by spraying with α-naphthol reagent in sulphuric acid, followed by heating until band development.

Comparative *in silico* chemotaxonomic analysis was performed using the annotated genomes of strain CPHL_1ᵀ and the reference strains *R. aureus* CMS 81y^T^ and *R. vestalii* LV3^T^. Genome annotations were retrieved from the NCBI database (https://www.ncbi.nlm.nih.gov/refseq). A targeted set of genes representative of major chemotaxonomic pathways was selected based on literature reports. Selected genes were further screened by blastp using representative sequences as queries, and hits were evaluated based on sequence identity, coverage and conserved domain architecture.

### 16S rRNA gene and whole-genome sequencing

Genomic DNA was extracted using the NucleoSpin Microbial DNA Mini kit (Macherey-Nagel) following the manufacturer’s protocol and quantified spectrophotometrically (6131 BioPhotometer, Eppendorf). The 16S rRNA gene was amplified using universal primers (8F/1517R, Eurofins Genomics) and sequenced by Eurofins Genomics. The resulting sequence was compared to the one extracted from the assembled genome using the ContEst16S tool to confirm accuracy [17]. Whole-genome sequencing was performed by MicrobesNG using the Illumina NovaSeq 6000 platform. Read quality control and adapter trimming were conducted with Trimmomatic (v0.30). *De novo* assembly was carried out with Unicycler (v0.5.0), and genome completeness was evaluated using CheckM [18, 19].

### Phylogenetic analyses

Phylogenomic analyses were carried out using the TYGS platform (https://tygs.dsmz.de) alongside TrueBacID (https://www.germai.app/) [20–22]. The fifteen closest type strains, based on 16S rRNA gene sequence similarity and available in the TYGS database, were selected for comparison. Reference genomes were retrieved from the NCBI database, and 16S rRNA gene sequences were extracted using the ContEst16S tool or recovered from the same database.

A phylogenetic tree based on 16S rRNA gene sequences was reconstructed using mega (v12) [23]. Multiple sequence alignment (MSA) was performed using muscle with the neighbour joining clustering method with two iterations [24]. Phylogeny inference was conducted using the maximum likelihood method and the Kimura two-parameter model [25, 26]. Tree topologies were re-examined by the bootstrap method of resampling using 1,000 replications [27].

A whole-genome-based phylogenetic tree was reconstructed using TYGS. Pairwise genome comparisons were performed using the Genome blast Distance Phylogeny (GBDP) approach, with intergenomic distances calculated using the ‘trimming’ algorithm and distance formula d5. Digital DNA–DNA hybridization (dDDH) values and confidence intervals were calculated using the recommended settings of the GGDC 4.0 [28]. The resulting intergenomic distances were used to infer a balanced minimum evolution tree with branch support via FASTME 2.1.6.1, including SPR postprocessing. Branch support was estimated from 100 pseudo-bootstrap replicates. The resulting tree was pruned and midpoint-rooted in R (v4.5.1) using packages ape (v5.8.1) and phytools (v2.5.2).

Average nucleotide identity (ANI) and dDDH values were calculated to assess genomic relatedness among the five closest strains identified. dDDH was obtained using JSpeciesWS (https://jspecies.ribohost.com/jspeciesws), while ANI was calculated using both MUMmer (for ANIm values) and blastn (for ANIb values) alignment algorithms [29].

### Genomic annotation and analyses

Genome annotation was performed using Prokka, and the locus tags were used for downstream analyses [30]. Functional annotations were compared with those obtained using BV-BRC (https://bv-brc.org), RAST (http://rast.nmpdr.org/) and KEGG (https://www.kegg.jp/) databases to refine gene predictions and pathway assignments [31–33]. Protein sequences were compared with those of related strains using the BV-BRC Proteome Comparison Tool (https://www.bv-brc.org/app/SeqComparison) through bidirectional blastp (minimum coverage 30%, E-value ≤1e-5, identity ≥10%). Biosynthetic potential was assessed using antiSMASH (v8.0) (https://antismash.secondarymetabolites.org/) with relaxed detection parameters [34].

### β-galactosidase recovery and biochemical characterization

β-Galactosidase activity was measured from crude cytosolic extracts of strain CPHL_1^T^. Cells were obtained from a 500 ml TSB culture inoculated (1 : 20) from a fresh culture (1×10^10^ c.f.u. ml^−1^) and incubated at 15 °C for 96 h at 90 r.p.m. Cells were harvested by centrifugation (8,000 x g, 20 min, 4 °C) and stored at −20 °C. Pellets were resuspended in 0.02 M phosphate buffer, pH 7.0 (0.30 g wet cells:0.90 ml buffer) and lysed through sonication on ice (600 W, 30 min) (Sonicator Ultrasonic Liquid Processor XL equipped with Microtip probe 3.1 mm, Heat Systems-Ultrasonic Inc.). The crude extract was recovered by centrifugation (15,000 x g, 10 min, 4 °C) and dialysed against the same buffer (cut-off size of 12–14 kDa dialysis tubes; Medicell membranes, 3 l of buffer changed every 15 h, three times). Total protein content was determined according to the Bradford method [35].

Enzymatic activity was assayed using chromogenic substrates (2-nitrophenyl *β*-d-galactopyranoside, 4-nitrophenyl *β*-d-galactopyranoside, 2-nitrophenyl *α*-d-galactopyranoside and 4-nitrophenyl *β*-d-lactopyranoside). Standard reactions (500 µl total volume) contained 5 mM substrate and 0.5 µg µl^−1^ total proteins in 0.02 M phosphate buffer, pH 7. Reactions were incubated at 4 °C for up to 90 min, and release of nitrophenol (NP) was quantified spectroscopically at λ420 nm. One unit of specific enzymatic activity (U mg^−1^) was defined as the activity corresponding to the release of 1 µmol of NP per mg of total proteins under these conditions [11].

The best-performing substrate (2 NP *β*-d-galactopyranoside) was used to determine optimal temperature and pH. Temperature dependence was assessed between 4–55 °C (20 min incubation), and pH dependence over the range pH 3–9 in the appropriate 0.02 M buffers (pH 3 citrate buffer (g l^−1^): Na_3_C_6_H_5_O_7_⋅2H_2_O 0.55, citric acid 3.48; pH 4 acetate buffer: CH_3_COONa 0.39, CH_3_COOH 0.93; pH 5 acetate buffer: CH_3_COONa 1.10, CH_3_COOH 0.93; pH 6 phosphate buffer: Na_2_HPO_4_⋅7H_2_O 0.73, NaH_2_PO_4_⋅H_2_O 2.38; pH 7 phosphate buffer; pH 8 phosphate buffer: Na_2_HPO_4_⋅7H_2_O 4.04, NaH_2_PO_4_⋅H_2_O 0.68; pH 9 Tris-HCl buffer: 2-amino-2-(hydroxymethyl)propane-1,3-diol 2.42). Thermostability was determined by pre-incubating extracts for 2 h at 4–35 °C before activity measurement at 35 °C. All assays were performed in triplicate, and mean values are reported.

### Identification, sequence analyses and phylogenetic analyses of β-galactosidases

The corresponding protein sequence of the β-galactosidase from strain CPHL_1^T^ was retrieved from the annotated genome in GenBank (accession number MHD2012605.1). Homologous sequences from *R. aureus* CMS 81y^T^ and *R. vestalii* LV3^T^ were identified using blastp searches against their respective predicted proteomes from the NCBI database [36, 37]. Hits showing full-length coverage and high sequence identity were selected for further analysis. Orthologous relationships were inferred based on high sequence identity (>85%) and complete alignment coverage. They were further confirmed by reciprocal blastp Reciprocal Best Hits analysis, whereby each candidate sequence was queried against the proteome of the originating strain, and the original sequence was recovered as the top hit.

Protein domain architecture and functional annotation were assessed using InterProScan (v108.0). MSA of the β-galactosidase sequences was performed using MAFFT (v7) with default parameters [38]. The resulting alignment was manually inspected using Jalview (v2.11.5.1), and conservation patterns were evaluated using built-in conservation scoring and consensus tools.

Protein sequences of β-galactosidases from the other *Rhodoglobus* strains and selected reference organisms were retrieved from the NCBI database. Sequences were aligned using MAFFT (v7). The final dataset consisted of 12 protein sequences aligned over 807 amino acid positions. Phylogenetic analysis was performed using the maximum-likelihood method implemented in IQ-TREE (v1.6.11) [39]. The best-fitting substitution model was selected automatically using ModelFinder, which identified LG+F+G4 as the optimal model. Branch support was assessed using the ultrafast bootstrap approximation (1,000 replicates) and the SH-aLRT test (1,000 replicates). The resulting tree was midpoint-rooted and visualized using iTOL (v7.5.1) [40].

## Results and discussion

### Morphological, physiological and biochemical characteristics

**Fig. 1.**
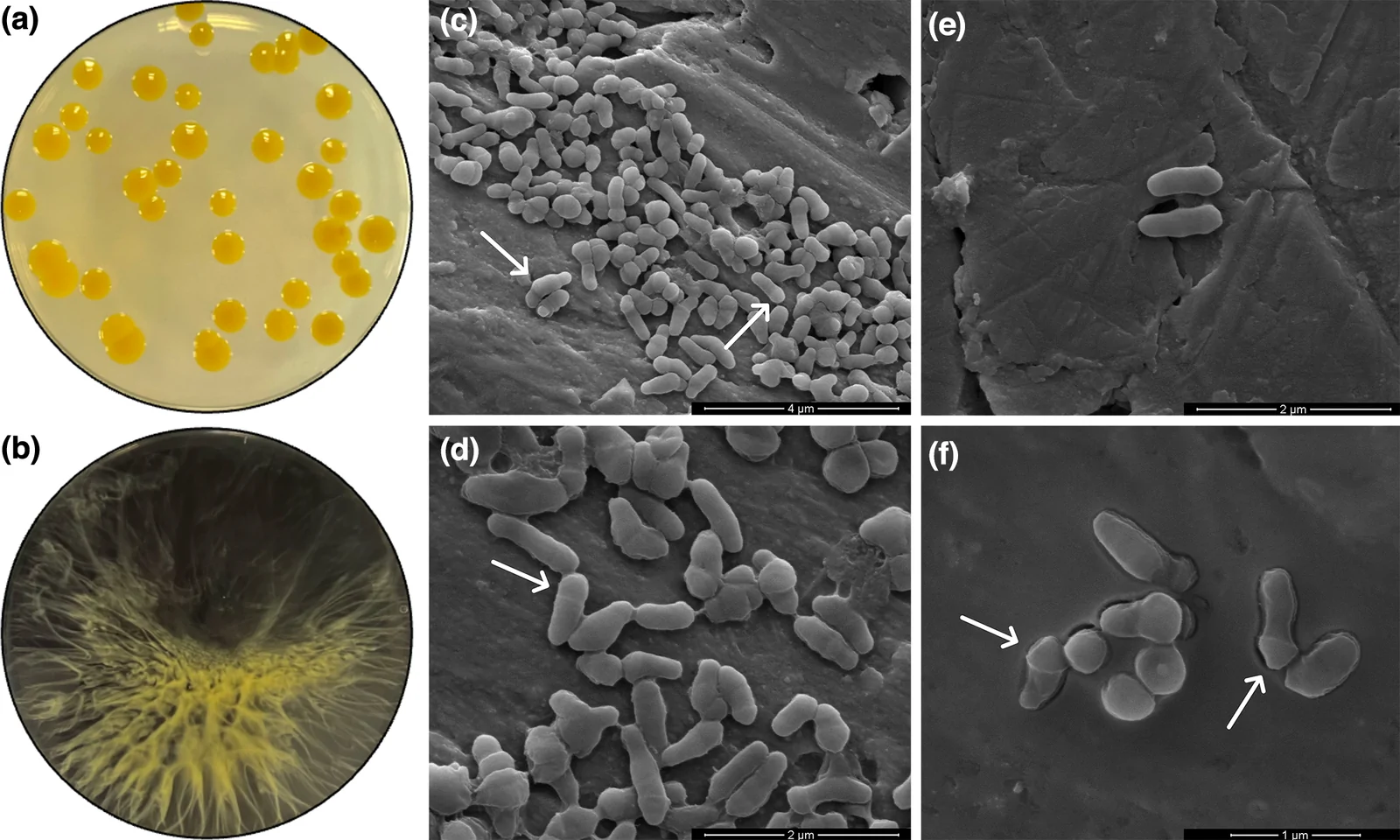
Colony morphology, biofilm formation and of *Rhodoglobus* sp. strain CPHL_1^T^ after 12 days at 15 °C. SEMs showing short rod-shaped cells with bulbous protuberances located at cell extremities central region.

Strain CPHL_1^T^ formed smooth, round, non-mucoid, yellow-orange colonies on solid medium (Fig. 1a). SEM observations were consistent with those previously reported for *R. vestalii* LV3^T^ [1]. Cells appeared as short rods, ∼0.2 µm in width and 0.7–1.0 µm in length (Fig. 1c–f). A characteristic swollen, bulbous structure was frequently observed at one cell extremity, measuring ∼0.3 µm in diameter and 0.2 µm in height. In contrast, globular protuberances located in the central region of the cells, previously described as predominant, were only rarely detected. The biological role of these structures remains unclear. As previously hypothesized, they may contribute to an increased surface-to-volume ratio, facilitating nutrient uptake, or they may reflect an atypical rod-coccus developmental cycle, as reported in several Gram-positive, non-spore-forming aerobic bacteria [1].

When grown in static liquid cultures, strain CPHL_1^T^ developed non-adherent, structured biofilms in modified R2A medium supplemented with 1% (w/v) sucrose (Fig. 1b). Growth occurred aerobically in TSB, LB and R2A media, with higher biomass yields observed in TSB and LB. Based on growth curves, the strain grew between 4 and 20 °C, with an optimum at 15 °C and a plateau up to 20 °C; however, increased cell aggregation in the broth was observed at higher temperatures, as previously reported for *R. vestalii* LV3^T^. No growth was detected at 35 °C, consistent with its psychrophilic nature. Growth at 4 °C was characterized by an extended lag phase. The strain grew at pH 7.0–9.0 (optimum 8.0) and tolerated up to 6% NaCl (optimum 0–3%) (Fig. S1).

Differential phenotypic characteristics compared with related *Rhodoglobus* species are summarized in Table 1. Similar to * R. aureus*, but in contrast to *R. vestalii*, strain CPHL_1^T^ reduced nitrate, suggesting a potential role in nitrogen cycling in Antarctic environments. As observed in both validly described members of the genus, the strain was catalase-positive, non-spore-forming and did not hydrolyse gelatin, cellulose, starch or casein under the tested conditions.

**Table 1.** Differential phenotypic and chemotaxonomic features of strain CPHL_1^T^ and the related *Rhodoglobus* species. Strains: 1, CPHL_1^T^, [this study]; 2, *R. vestalii* LV3^T^ [1]; 3, *R. aureus* CMS 81y^T^ [4, 5]. +, positive; w, weakly positive; -, negative; nd, not determined

	1	2	3
**Phenotypic and biochemical characteristic**			
Isolation source	Sediments	Cyanobacterial mat	Cyanobacterial mat
Colony colour	Yellow-orange	Red	Yellow
Cells’ length (µm)	0.7–1.0	1.0–1.2	nd
Temperature range (optimum)	4–20 °C (15 °C)	−2–21 °C (18 °C)	0–30 °C (22 °C)
pH range (optimum)	7.0–9.0 (8.0)	6.0–8.0 (7.0–7.02)	6.0–11.0 (7.0)
Salinity tolerance (optimum) (NaCl%, w/v)	0–6 (0–3)	0–10 (0–3)	0–3 (0)
Motility	−	+	−
Oxidase	w	nd	−
Nitrate reduction	+	−	+
**Chemotaxonomic analyses**			
Major menaquinones	MK-10, MK-11, MK-12	MK-11, MK-12	MK-11
Polar lipids	phosphatidylglycerol, diphosphatidylglycerol	nd	phosphatidylglycerol, diphosphatidylglycerol
Major fatty acids	anteiso-C_15 : 0_, anteiso-C_17 : 0_, iso-C_16 : 0_	anteiso-C_15 : 0_, anteiso-C_17 : 0_, iso-C_16 : 0_	anteiso-C_15 : 0_, anteiso-C_17:0,_ iso-C_16 : 0_
G+C content (%)	60	62	64

Carbon-source utilization assessed using GenIII MicroPlates showed the assimilation of a wide range of carbohydrates (including d-cellobiose, d-fructose, d-galactose, d-glucose, d-maltose, d-mannose, d-salicin, d-trehalose, d-turanose, and sucrose), polyols (d-arabitol, d-mannitol, d-sorbitol, glycerol, *myo*-inositol), and several amino acids (l-alanine, l-arginine, l-aspartic acid, l-glutamic acid, l-histidine). A complete list is provided in Table S1. Growth on modified R2A medium supplemented with 1% (w/v) of d-glucose, d-fructose, d-maltose, d-mannose, or sucrose confirmed these results. Despite methodological differences [4], several distinctions were observed between strain CPHL_1^T^ and *R. aureus* CMS 81y^T^, particularly in substrate utilization (e.g. d-cellobiose, d-maltose, d-melibiose, d-mannitol, d-sorbitol, *myo*-inositol, l-alanine, sucrose) and sensitivity to lincomycin. Conversely, both strains shared identical profiles for d-fructose, d-galactose, d-glucose, d-mannose, d-raffinose, lactose, l-arginine and rifampicin sensitivity. These phenotypic differences further support the assignment of strain CPHL_1^T^ as a distinct taxon within the genus *Rhodoglobus*.

### Chemotaxonomic characteristics

The predominant respiratory quinone was identified as MK-11 (50%); MK-10 (20%) and MK-12 (30%) were also detected. The major fatty acid methyl esters were anteiso-C_15 : 0_ (38.6%), anteiso-C_17 : 0_ (24.3%) and iso-C_16 : 0_ (17.3%), consistent with the fatty acid profiles previously reported for *R. vestalii* and *R. aureus*. Minor amounts of iso-C_17 : 0_ (9.5%), iso-C_15 : 0_ (8.6%), anteiso-C_16 : 0_ (0.9%) and C_14 : 0_ (0.4%) were also detected. In addition, the cell-wall sugars found were glucose, rhamnose and ribose (Fig. S2).

Intracellular pigment production during the exponential phase was consistent with other members of the family *Microbacteriaceae* [1]. Unlike *R. vestalii*, which forms red colonies, and *R. aureus*, which is yellow, strain CPHL_1^T^ displayed a distinct yellow-orange pigmentation. Crude methanol extracts of pigments showed absorbance peaks at λ 264, 448 and 476 nm, differing from those previously reported for *R. aureus* (Fig. S3). Differential chemotaxonomic characteristics compared with related *Rhodoglobus* species are summarized in Table 1.

### *In silico* chemotaxonomic features

Comparative genome analysis of strain CPHL_1ᵀ and the reference strains *R. aureus* CMS 81y^T^ and *R. vestalii* CMS 81y^T^ suggested the presence of a conserved set of genes associated with major chemotaxonomic pathways (Table 2). These genome-based predictions provide indirect evidence and are intended to complement the experimental chemotaxonomic characterization of strain CPHL_1^T^ [41].

**Table 2.** GenBank locus tags of genes involved in major chemotaxonomic pathways inferred from genome analysis. Strains: 1, CPHL_1^T^; 2, *R. vestalii* LV3^T^; 3, *R. aureus* CMS 81y^T^

Functional category	Gene	1	2	3
Fatty acid biosynthesis	*fabH*	ACTJJW_RS14635	ABD112_RS02790	FB472_RS01725
	*fabG*	ACTJJW_RS03065	ABD112_RS08825	–
Branched-chain fatty acid biosynthesis	*ilvE*	ACTJJW_RS04895	ABD112_RS13285	FB472_RS11990
Menaquinone	*menA*	ACTJJW_RS07320	ABD112_RS10500	FB472_RS05215
	*menB*	ACTJJW_RS07335	ABD112_RS10515	FB472_RS05230
	*menC*	ACTJJW_RS08895	–	–
	*menD*	ACTJJW_RS07305	ABD112_RS10485	FB472_RS05200
Peptidoglycan (DAP)	*murD*	ACTJJW_RS03655	ABD112_RS03240	FB472_RS01255
	*murE*	ACTJJW_RS06600	ABD112_RS00765	FB472_RS04650
	*dapA-D, dapF*	(present)	(present)	(present)
Cell wall sugars	*galE*	ACTJJW_RS04590; RS14400	ABD112_RS00805; RS13550	FB472_RS03095; RS11695
	*galU*	ACTJJW_RS04330	ABD112_RS12900	FB472_RS11440

All genomes encoded key components of the type II fatty acid synthesis pathway, including *fabH* or *fabG*, suggesting the presence of a canonical fatty acid biosynthetic system. However, no clear homologues of *fabF* or FabI-type enoyl-acyl carrier protein reductases were identified. Several short-chain dehydrogenase/reductase family proteins were detected, but could not be confidently assigned to this function, indicating either highly divergent homologues or alternative enzymatic systems [42].

All strains possessed *ilvE*, encoding branched-chain amino acid aminotransferase involved in the formation of α-keto acid precursors from valine, leucine and isoleucine. In contrast, homologues of the branched-chain α-keto acid dehydrogenase complex (*bkd* operon) were not identified, suggesting a limited genomic basis for a complete branched-chain fatty acid biosynthetic pathway [42].

Genes involved in menaquinone biosynthesis, including *menA* and *menB*, were consistently detected, with *menC* and/or *menD* variably present, indicating a conserved but partially variable capacity for quinone biosynthesis [43]. All genomes encode key genes of peptidoglycan biosynthesis and the diaminopimelate pathway, including *murE*, *murD* and multiple *dap* genes (*dapA*, *dapB*, *dapC*, *dapD* and *dapF*), supporting a DAP-type cell wall [44]. In addition, *galE* and *galU* were identified, suggesting a potential nucleotide sugar interconversion pathway involved in cell wall polysaccharide biosynthesis [45]. Overall, these *in silico* chemotaxonomic analyses provide supportive evidence consistent with the experimentally determined chemotaxonomic profile of strain CPHL_1^T^, reinforcing its assignment to the genus *Rhodoglobus*.

### 16S rRNA gene and genome sequences

Phylogenetic analyses based on 16S rRNA gene sequences placed strain CPHL_1^T^ within the genus *Rhodoglobus*, showing high similarity to *R. vestalii* LV3^T^ (99.4%) and *R. aureus* CMS 81y^T^ (97.6%).

The draft genome of strain CPHL_1^T^ comprised 2.9 Mb assembled in 38 contigs, with a G+C content of 60%, consistent with other members of the genus [1, 4, 5]. The assembly showed high quality, with an N50 of 316.3 kb, an L50 of 4 and CheckM-estimated completeness and contamination of 99.08 and 0.88%, respectively. A total of 2847 protein-coding sequences were predicted, of which ∼96% were assigned putative functions, while the remaining were annotated as hypothetical proteins. The genome also contained 45 tRNA genes and 5 rRNA genes, and it was associated with 250 functional subsystems.

**Fig. 2.**
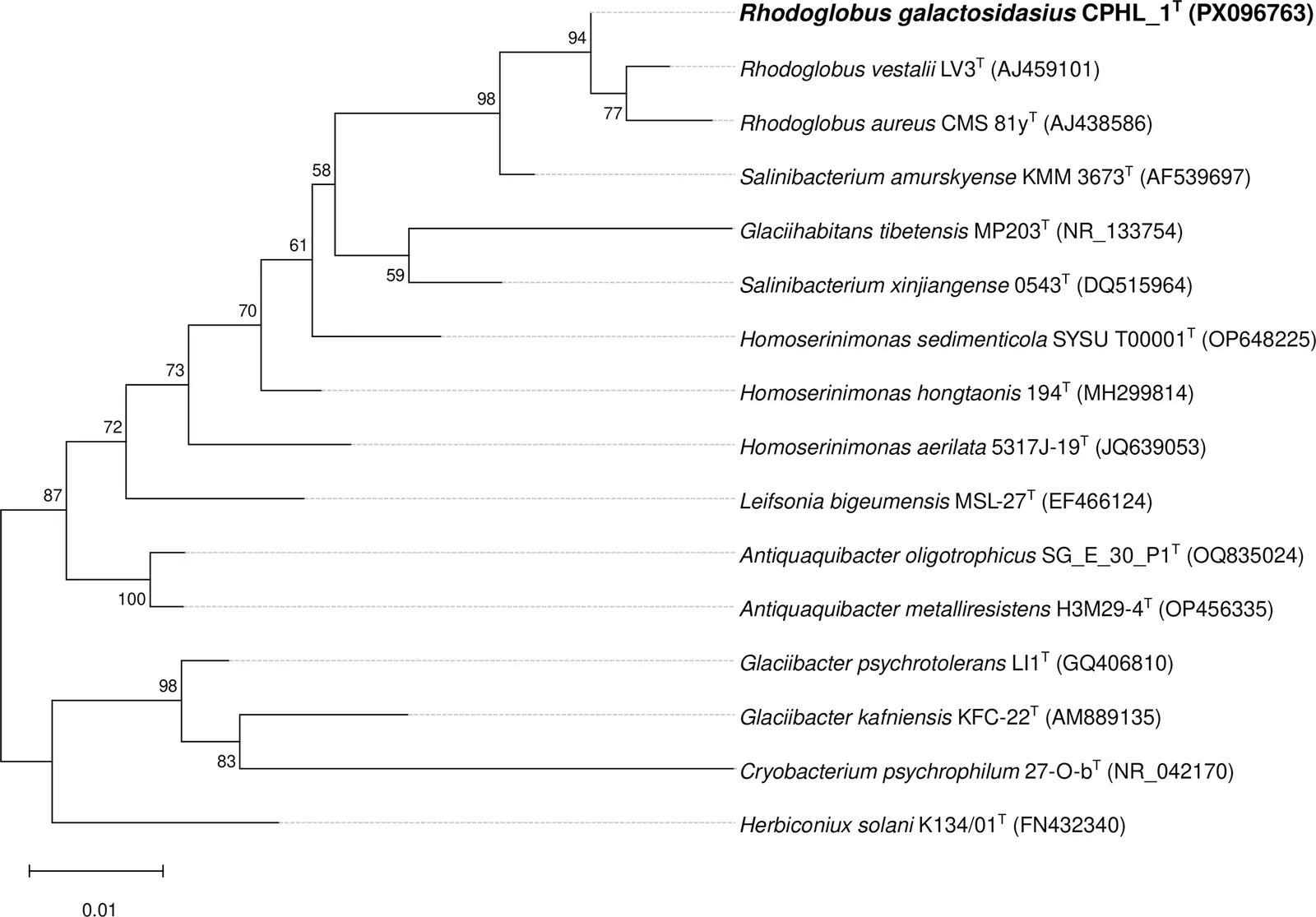
Maximum-likelihood phylogenetic tree based on 16S rRNA gene sequences showing the position of strain CPHL_1^T^ within the genus *Rhodoglobus*. Sequences were aligned using muscle, and the tree was reconstructed using the Kimura two-parameter model. Bootstrap values (>50%, based on 1,000 replicates) are shown at branch nodes. GenBank accession numbers are indicated in parentheses.

**Fig. 3.**
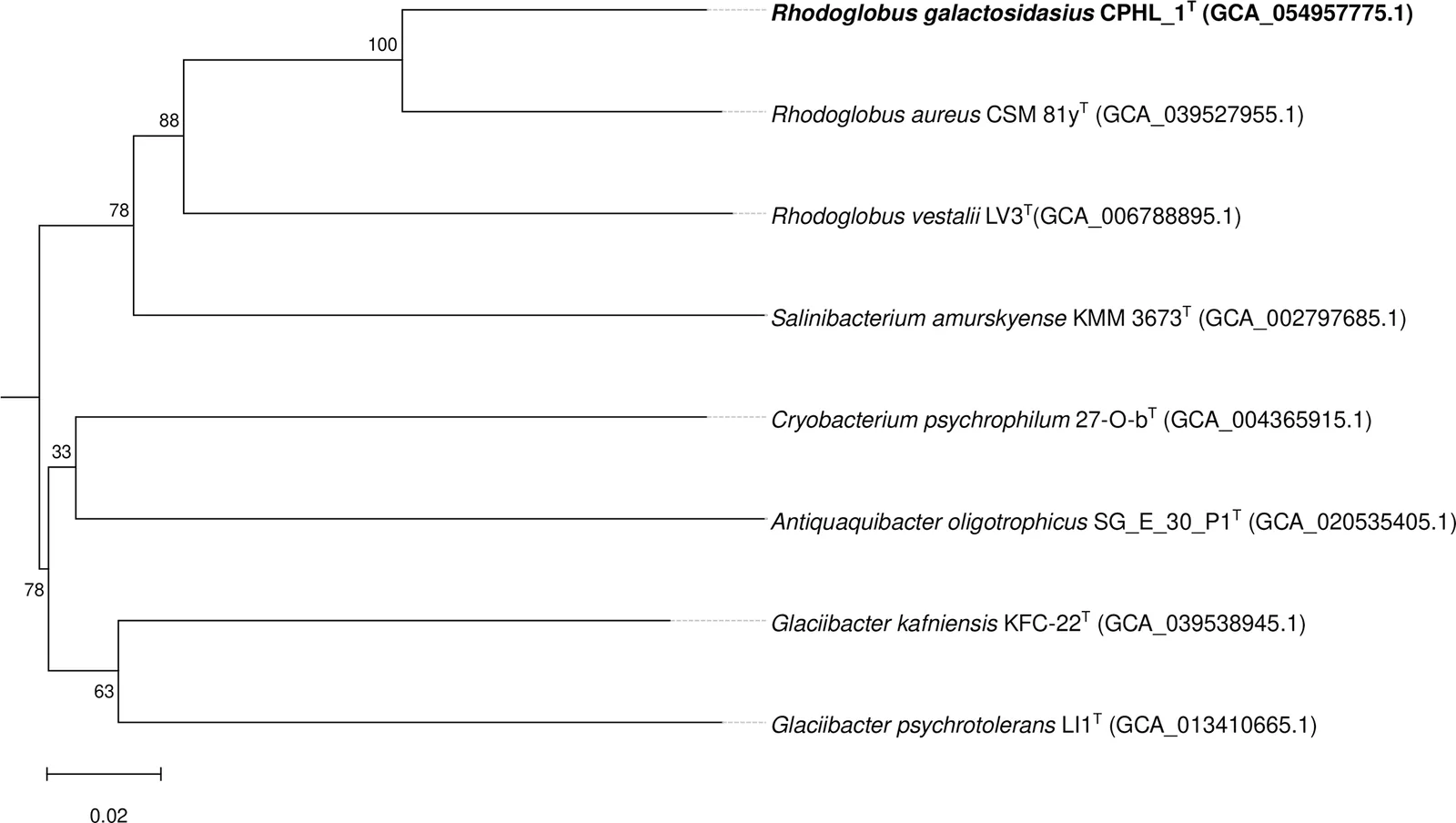
Genome-based phylogenetic tree showing the position of strain CPHL_1^T^ inferred using the GBDP implemented in TYGS. The tree was reconstructed with FASTME 2.1.6.1 using the GBDP distance formula d5. Branch lengths correspond to GBDP distances. Numbers at nodes indicate pseudo-bootstrap support values based on 100 replications. GenBank accession numbers are indicated in parentheses.

To clarify the taxonomical placement of strain CPHL_1^T^, its genome was compared with those of fifteen closely related type strains. Both 16S rRNA gene-based phylogeny (Fig. 2) and whole-genome GBDP distances consistently positioned the strain within the genus *Rhodoglobus*, with clear separation from its closest relatives (Fig. 3). Genome-based phylogeny provided higher resolution than the 16S rRNA gene-based tree, with *R. aureus* JCM 12762^T^ identified as the closest relative. Genomic similarity metrics supported this classification: dDDH values were below the 70% species threshold [28], with a maximum of 38.3% vs * R. aureus* ^T^, and ANI values below the 95% species cutoff (ANIb 88.9%, ANIm 90.0%) (Table 3) [46]. Comparisons with related genera, such as *Cryobacterium* and *Salinibacterium* yielded substantially lower ANI and dDDH values, as well as reduced total genome alignments, reinforcing the affiliation of strain CPHL_1^T^ to the genus *Rhodoglobus*.

**Table 3.** Genomic relatedness between strain CPHL_1^T^ and closely related type strains. dDDH values are reported with confidence intervals. ANI values were calculated using blastn (ANIb) and MUMmer (ANIm), with percentages of aligned nucleotides indicated in brackets

Genomes compared	Accession no.	dDDH [confidence interval] (%)	ANIb [aligned nucleotide] (%)	ANIm [aligned nucleotide] (%)
*R. aureus* CSM 81y^T^	GCA_039527955.1	38.3 [35.9–40.8]	88.9 [70.8]	90.0 [71.3]
*R. vestalii* LV3^T^	GCA_006788895.1	25.1 [22.8–27.6]	81.7 [60.4]	84.7 [44.0]
*Salinibacterium amurskyense* KMM 3673^T^	GCA_002797685.1	23.0 [20.7–25.5]	79.8 [59.7]	84.5 [36.0]
*Cryobacterium psychrophilum* 27-O-b^T^	GCA_004365915.1	23.8 [21.5–26.3]	70.1 [36.9]	86.1 [3.4]
*Antiquaquibacter oligotrophicus* SG_E_30_P1^T^	GCA_020535405.1	19.8 [17.6–22.2]	70.5 [36.3]	84.0 [2.6]
*Glaciibacter kafniensis* KFC-22^T^	GCA_039538945.1	20.0 [17.8–22.4]	69.5 [21.4]	83.8 [1.7]
*Glaciibacter psychrotolerans* LI1^T^	GCA_013410665.1	21.2 [19.0–23.6]	69.5 [26.7]	84.4 [1.8]

Proteome comparison analysis yielded consistent results, further supporting the delineation of strain CPHL_1^T^ as a novel species within this genus (Fig. S4).

### Genomic insights into metabolism, cold adaptation and secondary metabolite biosynthesis

KEGG annotation assigned 49.9% of predicted proteins to known pathways, and the most represented categories included carbohydrate metabolism (12.5%), genetic information processing (10.9%), signalling and cellular processes (10.8%) and environmental information processing (10.6%).

The psychrophilic nature of strain CPHL_1^T^ was supported by the presence of genes associated with cold adaptation, including cold-shock proteins and RNA helicases involved in low-temperature responses [47, 48]. Genes related to fatty acid metabolism, including a fatty acid oxidation complex subunit α, may contribute to membrane fluidity regulation (Table S2).

Several carbohydrate-active enzymes were identified, including glycoside hydrolases (EC 3.2.1), among which a β-galactosidase (EC 3.2.1.23) was experimentally confirmed. Additional enzymes included maltodextrin glucosidase (EC 3.2.1.20), oligo-1,6-glucosidase (EC 3.2.1.10), β-hexosaminidase (EC 3.2.1.52), two trehalases (EC 3.2.1.28) and two bifunctional β-d-glucosidase/β-d-fucosidases (EC 3.2.1.21) (Table S2).

Consistent with its affiliation to *Actinomycetota*, the genome encodes pathways for carotenoid biosynthesis [49]. Key enzymes included phytoene desaturase (EC 1.3.99.29) and isopentenyl diphosphate isomerase (EC 5.3.3.2), potentially enhancing flux toward C_40_ carotenoids [50]. A polyketide synthase-like (Psi11) was also identified, suggesting the potential for α-pyrone synthesis, which is associated with polyketide pigments (Table S2) [51]. This is consistent with the metabolic potential of rare *Actinomycetota* members often overlooked by conventional isolation methods, which are increasingly recognized as sources of secondary metabolites with biotechnological potential [52].

AntiSMASH analysis revealed multiple biosynthetic gene clusters, including those for a terpene precursor and β-lactone (node 2), a terpene (node 6), a ribosomally synthesized and post-translationally modified peptide-like (node 8), a type III polyketide synthase (node 10) and a butyrolactone (node 11). Some clusters showed similarity to known pathways, such as the terpene cluster (62% similarity with a carotenoid cluster from *Dietzia* sp. CQ4), the type III polyketide synthases cluster (71% similarity with a benzoquinone cluster from *Streptomyces bikiniensis* subsp. *griseus* C1) and the butyrolactone cluster (77% similarity with a cluster from *Streptomyces coelicolor* A3(2)).

Overall, these genomic features highlight the metabolic versatility and environmental resilience of the genus *Rhodoglobus*, reinforcing the biotechnological potential of strain CPHL_1^T^ for low-temperature biotechnological applications.

### Cold-active β-galactosidase activity

Cytosolic crude extracts of strain CPHL_1^T^ exhibited β-galactosidase activity when tested with 2-nitrophenyl *β*-d-galactopyranoside and 4-nitrophenyl *β*-d-galactopyranoside, while no activity was detected against 2-nitrophenyl *α*-d-galactopyranoside or 4-nitrophenyl *β*-d-lactopyranoside (Fig. S5A). Release of 2-NP and 4-NP from the synthetic substrates revealed the presence of a *β*-galactosidase activity able to catalyse the hydrolysis of a *β*-glycosidic bond between C1 of galactose and *O* of the phenolic group. Conversely, the assay conditions did not reveal any *α*-galactosidase activity. No activity was detected with 4-nitrophenyl *β*-d-lactopyranoside and it may be related to the absence of a *β*-glucosidase activity able to free the 4-NP group from the glucose in the structure.

The highest specific activity toward 2-nitrophenyl *β*-d-galactopyranoside was observed at 35 °C (0.035 U mg^−1^). Notably, the enzyme showed 73.7±6.7% and 63.4±5.6% of its maximal activity at 15 °C and 4 °C, respectively, confirming its cold-active nature (Fig. S5B). The enzyme displayed a pH optimum of 7.0, maintaining >80% activity between pH 5.0 and 8.0 (Fig. S5C). Thermostability assays revealed that pre-incubation at≤25 °C for 2 h preserved activity, whereas exposure at 35 °C resulted in near-complete inactivation (Fig. S5D).

Genome annotation identified a putative *β*-galactosidase gene (EC 3.2.1.23; 2040 bp; locus tag 00328). While *β*-galactosidase activity has been previously reported in *R. vestalii* and *R. aureus*, and in the case of *R. vestalii* it was suggested to be cold-active, detailed characterization was lacking [4]. The properties observed here extend the known enzymatic potential of the genus, particularly for cold-processing applications, such as low-temperature hydrolysis in dairy industries [53].

### Comparative analysis of *β*-galactosidases in the genus *Rhodoglobus*

The *β*-galactosidase sequences from the three *Rhodoglobus* strains exhibited high pairwise sequence identity and aligned over their full length, supporting their orthologous relationship. The enzyme from strain CPHL_1^T^ showed the highest similarity to *R. aureus* (93.4%), while a slightly lower identity value was observed with *R. vestalii* (86.8%).

Functional annotation using InterProScan assigned all sequences to glycoside hydrolase family 42 (GH42), with a conserved domain architecture comprising an N-terminal GH42 domain, a β-galactosidase C-terminal domain and a trimerization domain. No additional domains or structural rearrangements were detected between them, indicating a highly conserved overall organization.

**Fig. 4.**
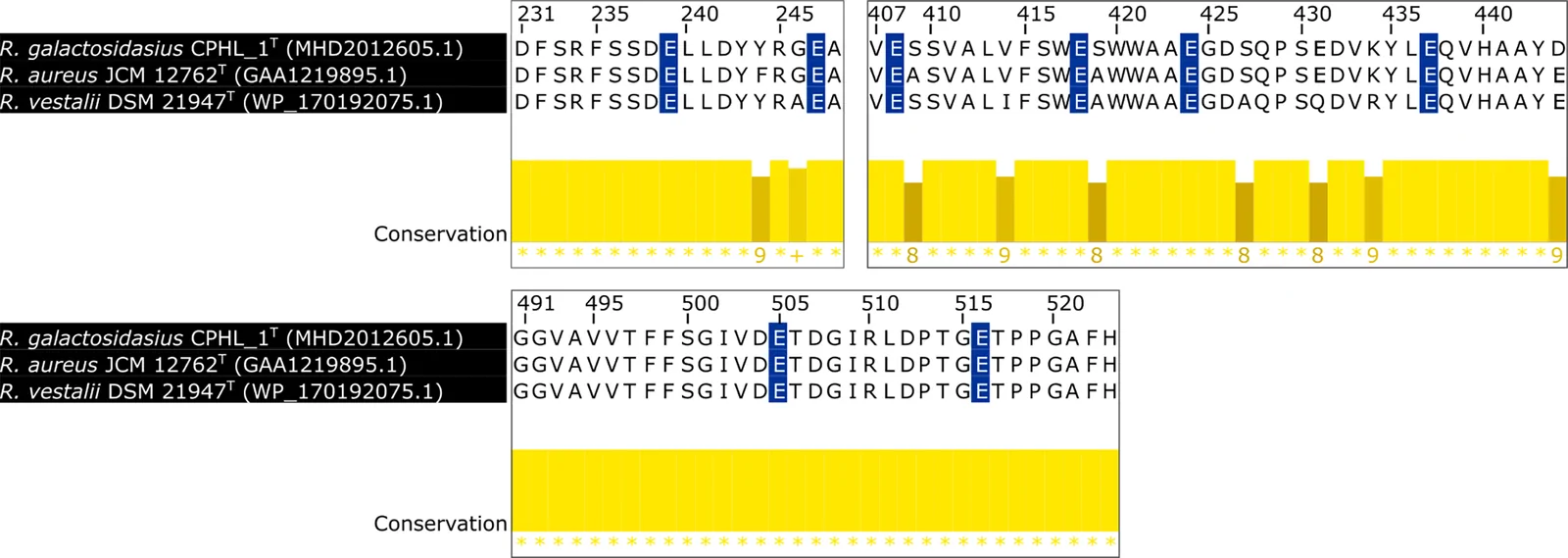
Partial MSA of GH42 β-galactosidases from the genus *Rhodoglobus*. Conserved regions are highlighted across the three sequences. Invariant Glutamate (E) residues are shown in blue, while conservation scores are indicated in yellow. The alignment is based on sequences aligned using MAFFT, and residue numbering refers to the alignment positions with the CPHL_1^T^ sequence used as reference. GenBank accession numbers are indicated in parentheses.

MSA performed with MAFFT revealed a high degree of conservation across the three β-galactosidases, particularly within several discrete regions distributed along the sequence (Fig. 4). These conserved regions are likely associated with structural stability and catalytic function, whereas more variable segments may contribute to functional fine-tuning or environmental adaptation, as commonly observed in GH families [54, 55]. Among the conserved regions, a central segment containing the motifs DFSRFSSDELLDYYRGEA showed strong conservation of acidic residues, including invariant glutamate (E) residues. Additional conserved blocks were identified in downstream regions enriched in glycine and acidic residues. The presence of multiple conserved glutamate residues within these regions is consistent with their proposed role in catalysis, in agreement with the established mechanism of GH42 β-galactosidases, which typically involves two glutamate residues acting as catalytic nucleophile and acid/base [56, 57].

### Phylogenetic relationships of GH42 β-galactosidases

**Fig. 5.**
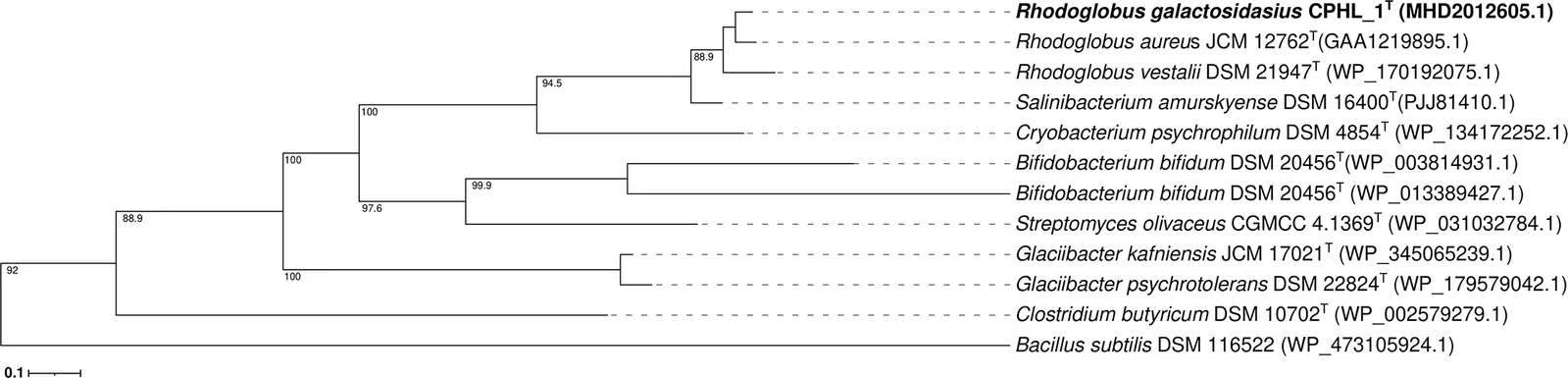
Maximum-likelihood phylogenetic tree of GH42 β-galactosidases inferred using IQ-TREE under the LG+F+G4 substitution model. The analysis was based on 12 protein sequences and 807 aligned amino acid positions. Branch support values are indicated as SH-aLRT/UFBoot percentages. The tree was midpoint-rooted. GenBank accession numbers are indicated in parentheses.

Phylogenetic analysis based on maximum-likelihood inference revealed that the β-galactosidases from the three *Rhodoglobus* strains form a well-supported clade, consistent with their taxonomic relatedness and high sequence similarity (Fig. 5). This clustering supports the conservation of GH42 β-galactosidases within the genus and suggests a shared evolutionary origin. More distantly related sequences formed separate clades corresponding to their taxonomic affiliations, providing a broader evolutionary framework for the analysed enzymes.

### Description of *Rhodoglobus galactosidasius* sp. nov

*R. galactosidasius* (ga.lac.to.si.da’si.us. N.L. masc. adj. *galactosidasius*, pertaining to β-galactosidase production).

Cells are Gram-positive, aerobic, non-motile, non-spore-forming short rods (0.7–1.0 µm long and ∼0.2 µm wide), occasionally bearing bulbous protuberances. Colonies are yellow-orange, circular, smooth and convex. Growth occurs at 4–20 °C (optimum 15 °C), pH 7.0–9.0 (optimum pH 8.0) and 0–6% (w/v) NaCl (optimum 0–3). The strain is catalase-positive and weakly oxidase-positive and does not hydrolyse gelatin, cellulose, starch or casein.

The predominant respiratory quinones are MK-11, MK-10 and MK-12, the major polar lipids are phosphatidylglycerol and diphosphatidylglycerol, and the cell-wall sugars are glucose, rhamnose and ribose. The major fatty acid methyl esters are anteiso-C_15 : 0_, anteiso-C_17 : 0_ and iso-C_16 : 0_.

The type strain is CPHL_1^T^ (=ITEM 19986^T^ =KCTC 59592^T^), isolated from coastal sediment at Cape Hallett, Antarctica (Antarctic Specially Protected Area no. 106). The genomic G+C content is 60%. The GenBank accession numbers are PX096763 (16S rRNA gene) and JBQIAV000000000 (whole-genome sequence).

## Supplementary material

10.1099/ijsem.0.007263 Supplementary Material 1.

## Abbreviations

ANI: average nucleotide identity
dDDH: digital DNA-DNA hybridization
ESI-MS: electrospray ionization mass spectrometry
GBDP: genome BLAST distance phylogeny
GH42: glycoside hydrolase family 42
LB: Luria Bertani
MSA: multiple sequence alignment
NP: nitrophenol
SEM: scanning electron microscopy
TSB: tryptic soy broth
