# Supplementary material for: Rhodoglobus galactosidasius sp. nov., a psychrophilic Actinomycetota producing a cold-active β-galactosidase isolated from Cape Hallett, Antarctica
Source: Int J Syst Evol Microbiol. 2026 Sep 16;76(9):007263. doi: 10.1099/ijsem.0.007263 (PMC13585282; doi:10.1099/ijsem.0.007263)
Supplement: Supplementary file 1 [file ijsem-76-07263-s001.pdf]

Supplementary Material

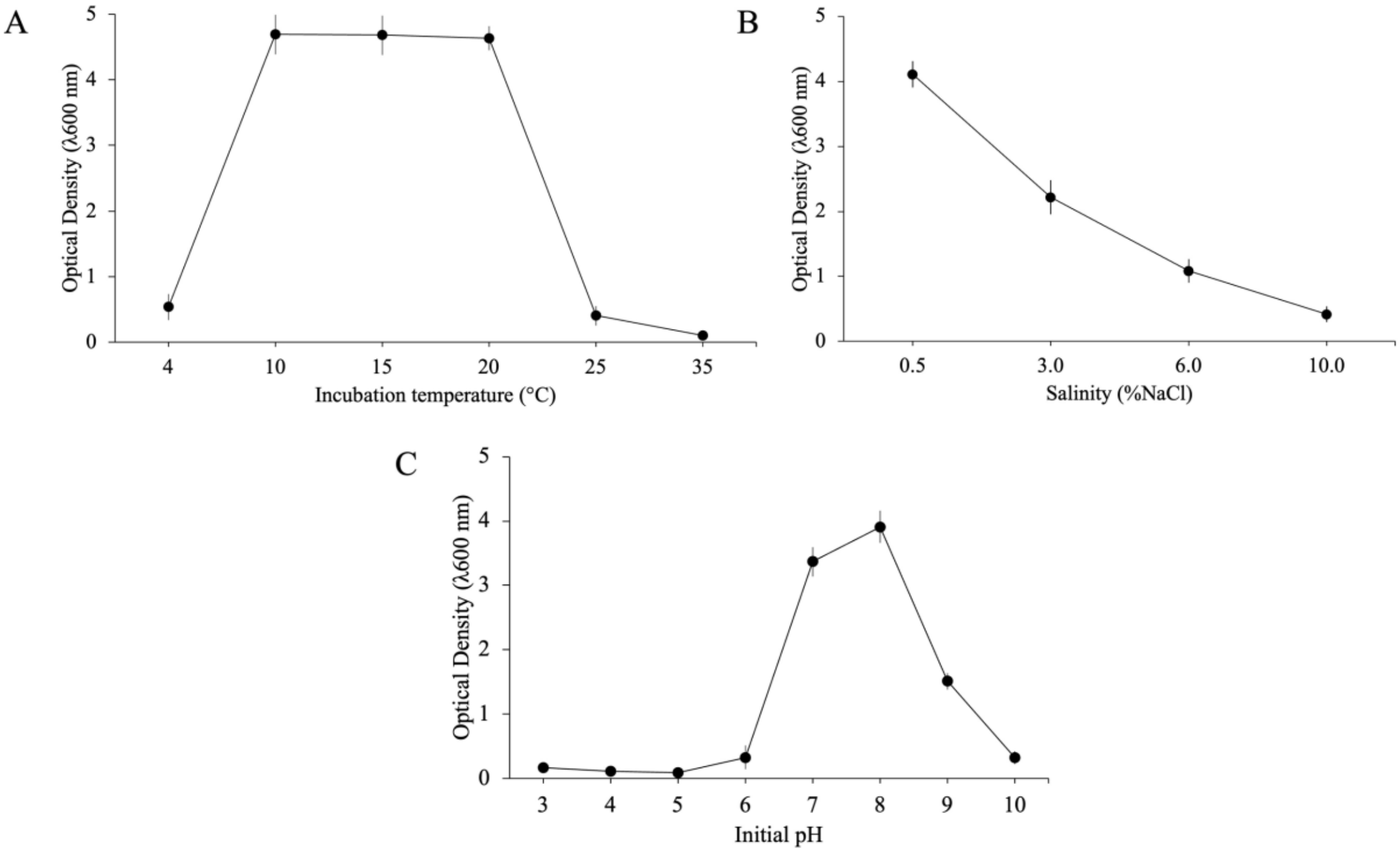

Supplementary Figure S1. **Growth curves of *Rhodoglobus galactosidasius* CPHL_1^{T}**. (A) Growth at different temperatures (°C), (B) NaCl concentrations (%), (C), and pH values. Optical density measured at λ 600 nm after 96 h incubation. Mean ± SD of biological triplicates.

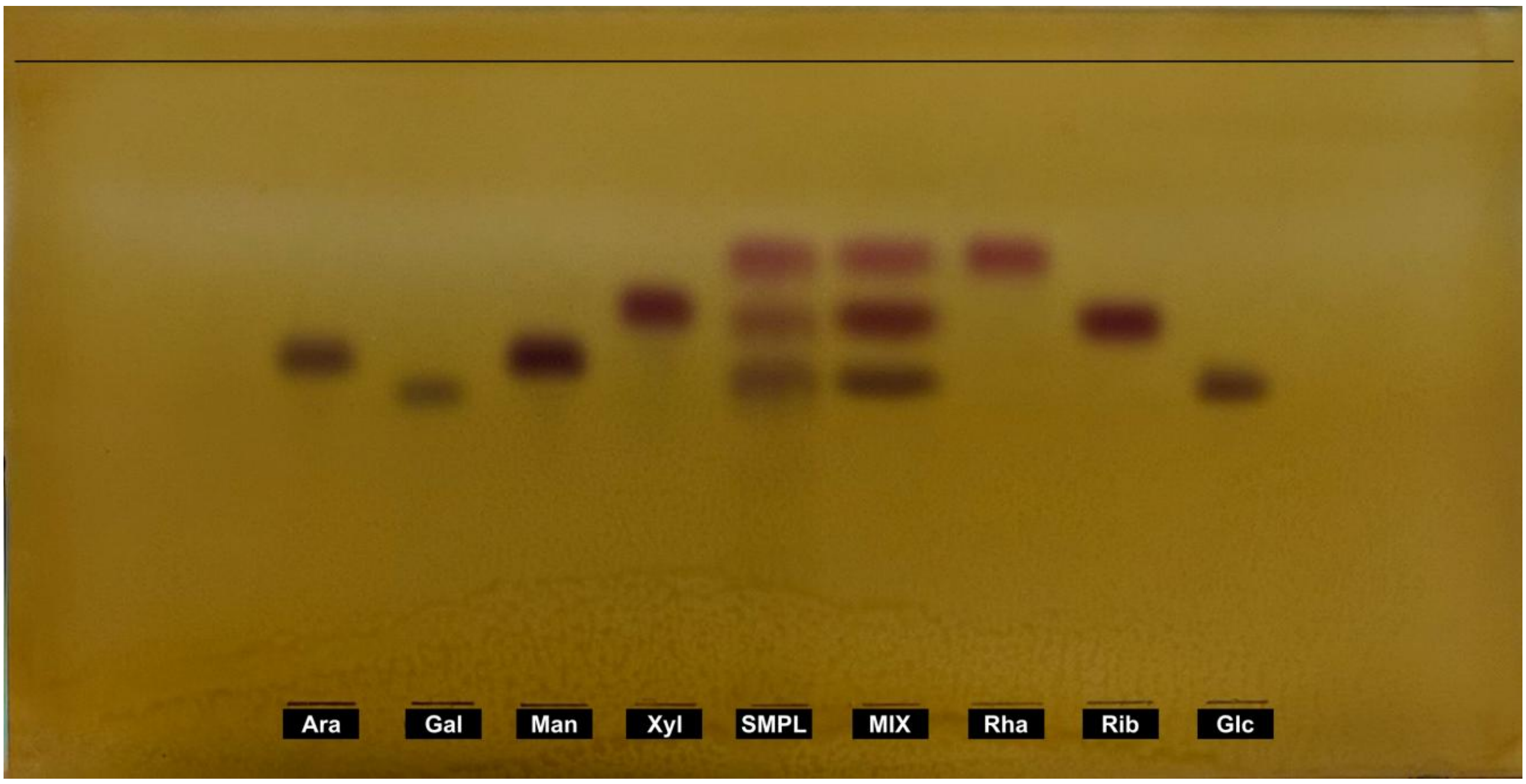

Supplementary Figure S2. **TLC analysis of cell-wall monosaccharides of *Rhodoglobus galactosidasius* CPHL_1^{T}.** Cell wall hydrolysates (SMPL) were analyzed by TLC on silica gel 60 F_{254} plates (10 x 20 cm). Monosaccharide standards (1 mg/mL solutions; Ara, Gal, Man, Xyl, Rha, Rib, Glc) and a sugar mix (Rha, Rib, Glc; MIX) were included for comparison. Separation was

performed with ethyl acetate/acetic acid/2-propanol/formic acid/water (25:10:5:1:15, v/v/v/v/v) for 1 h. Spots were visualized using α-naphthol reagent followed by heating.

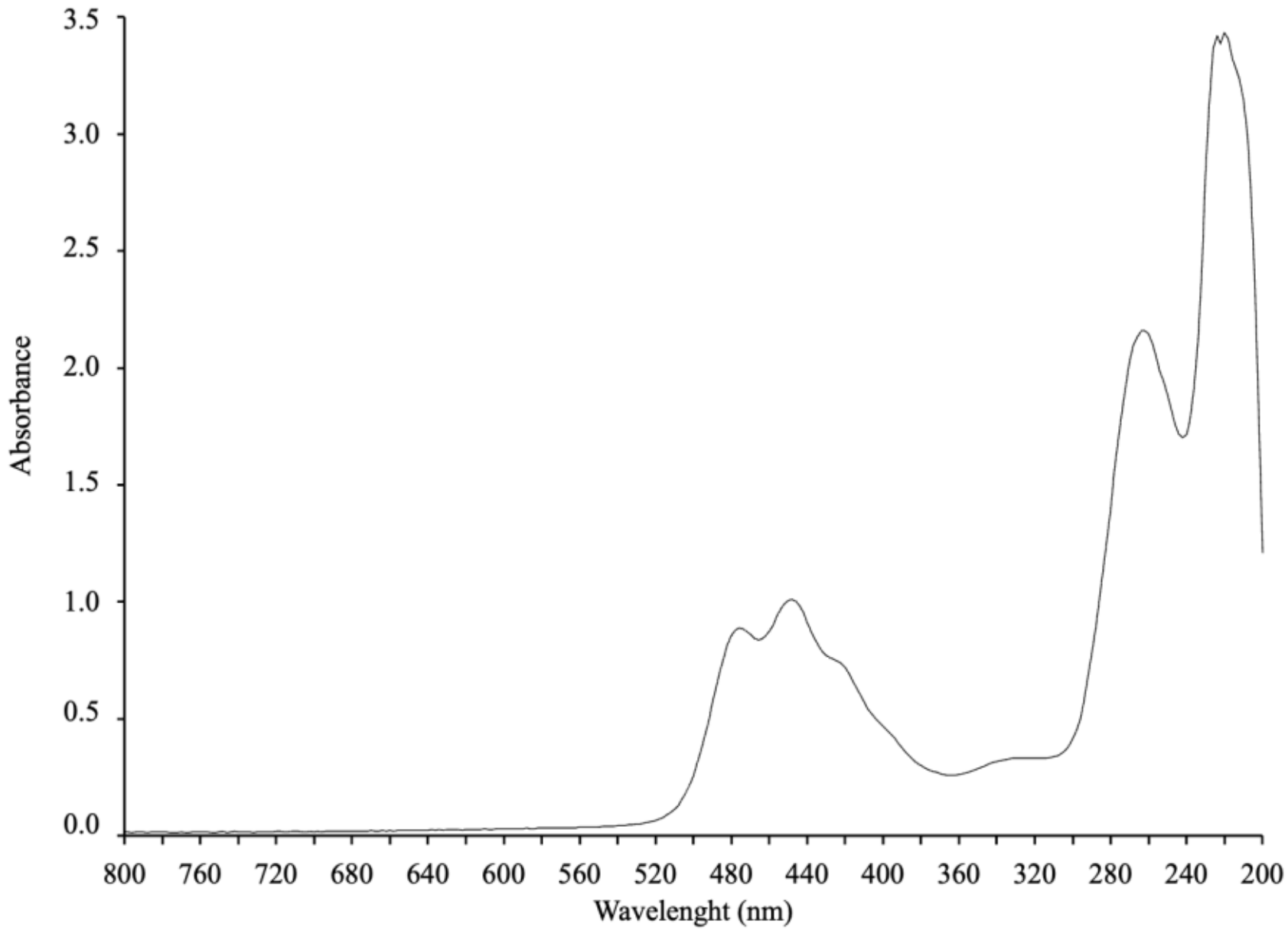

Supplementary Figure S3. **UV-Vis spectrum of pigment extracts of *Rhodoglobus galactosidasius* CPHL_1^T**. Absorbance of crude methanolic extract measured between λ200-800 nm; peaks at λ264, 448, and 476 nm. Methanol was used as a blank.

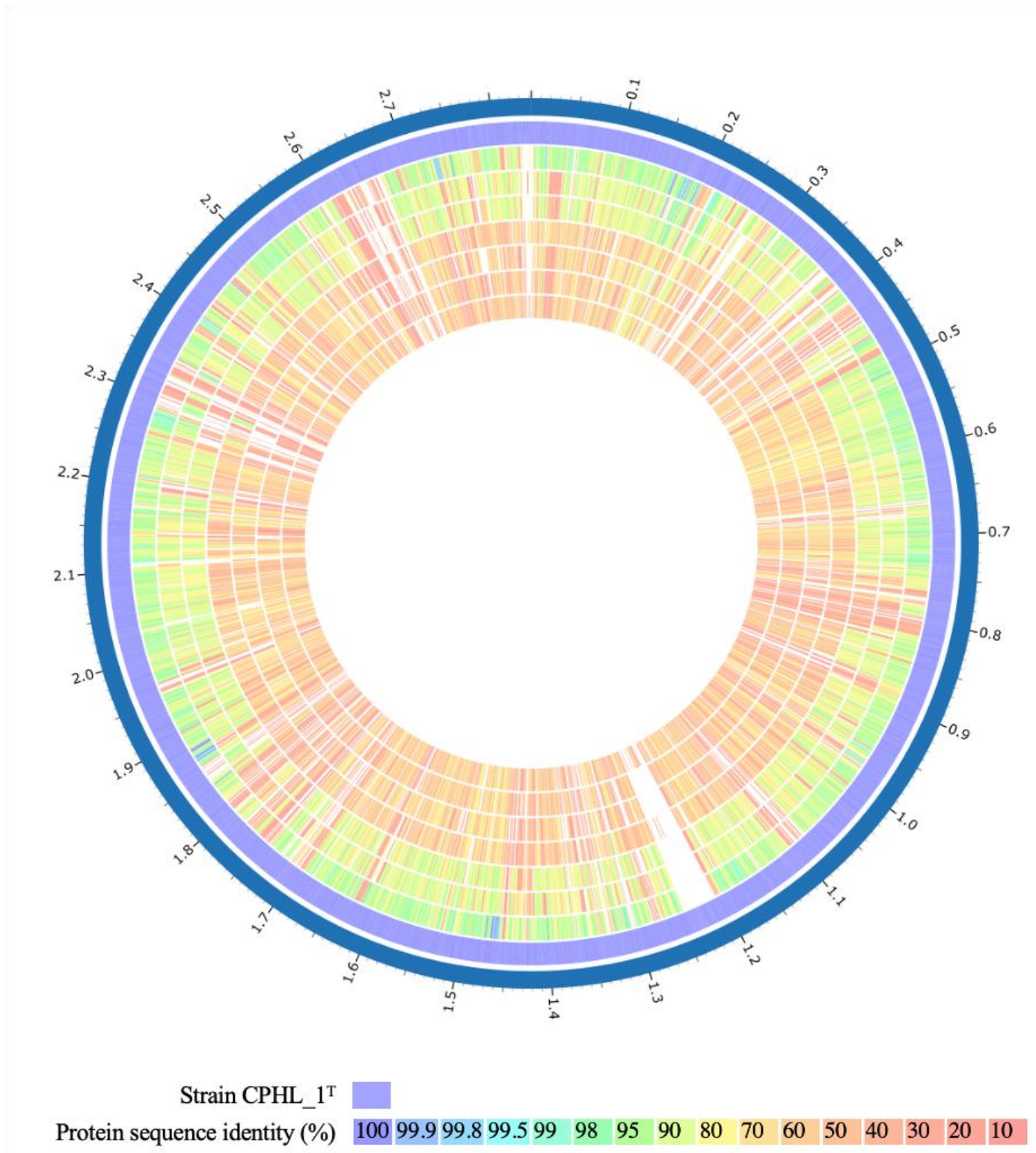

Supplementary Figure S4. **Whole-proteome comparison of *Rhodoglobus galactosidasius* CPHL_1^T and seven related type strains**. Circular plot displaying bidirectional protein sequence identity (dark blue: 100%; red: 10%). List of tracks, from outside to inside: strain CPHL_1^T,

Rhodoglobus aureus JCM 12762^T, *Rhodoglobus vestalii* DSM 21947^T, *Salinibacterium amurskyense* DSM 16400^T, *Cryobacterium psychrophilum* DSM 4854^T, *Antiquaquibacter oligotrophicus* DSM 111415^T, *Glaciibacter kafniensis* JCM 17021^T, *Glaciibacter psychrotolerans* DSM 22824^T.

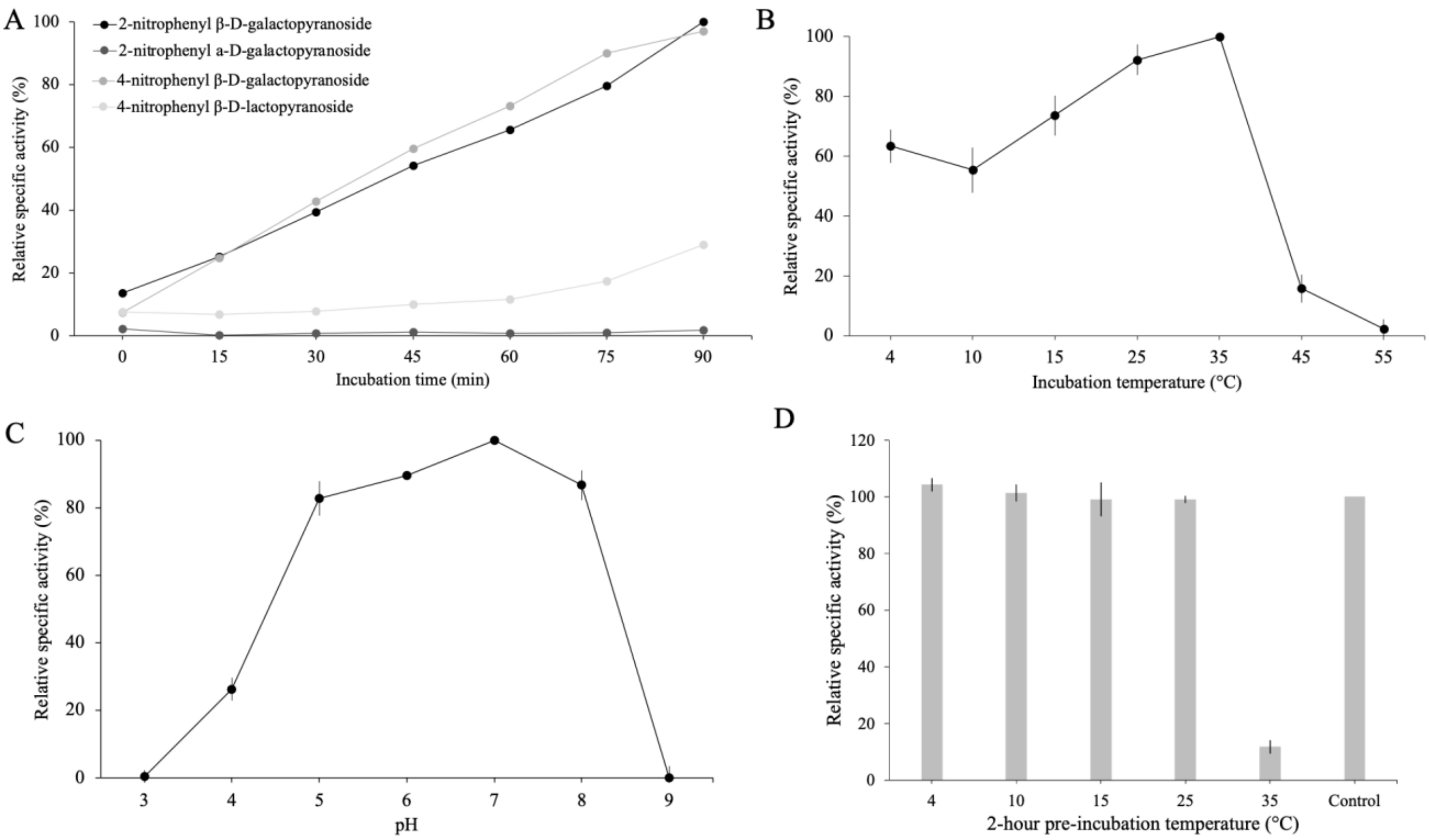

Supplementary Figure S5. **Spectrophotometric enzymatic assays of the crude extract of the cytosolic fraction of *Rhodoglobus galactosidasius* CPHL_1^T**. (A) Time course hydrolysis of different chromogenic substrates tested at 4 °C. (B) Temperature optimum for 2-nitrophenyl β-D-galactopyranoside. (C) pH optimum for 2-nitrophenyl β-D-galactopyranoside. (D) Thermostability for 2-nitrophenyl β-D-galactopyranoside. One unit of specific enzymatic activity was defined as the activity releasing 1 μmol of 2- and 4-nitrophenol by 1 mg of total proteins under the described assay conditions. Assays B, C, and D were performed in triplicate, and mean values are reported.

Supplementary Table S1. **Carbon source utilization and chemical sensitivity of *Rhodoglobus galactosidasius* CPHL_1**^{T} **assessed by Biolog GENIII MicroPlate**. Assays performed as in triplicate. +, positive; -, negative.

**Sugars**		D-Saccharic Acid	+	α-Hydroxy-Butyric Acid	-	Fusidic Acid	-
1% Sodium Lactate	-	L-Galactonic Acid Lactone	+	α-Keto-Butyric Acid	-	Lincomycin	-
D-Cellobiose	+	Pectin	+	α-Keto-Glutaric Acid	+	Macrolide	-
D-Fructose	+	**Carboxylic, aromatic and fatty acids**		β-Hydroxy-D,L-Butyric Acid	-	Minocycline	-
D-Fucose	-	Citric Acid	-	**Amino acids/Derivatives**		Rifamycin SV	-
D-Galactose	+	D-Lactic Acid Methyl Ester	+	D-Aspartic Acid	-	Vancomycin	-
D-Maltose	+	L-Lactic Acid	+	D-Serine	-	**Salts/Oxidizers/Inorganics**	
D-Mannose	+	Methyl Pyruvate	+	Glycyl-L-Proline	+	Potassium Tellurite	-
D-Melibiose	-	*p*-Hydroxy-Phenylacetic Acid	-	L-Alanine	+	Lithium Chloride	+
D-Raffinose	-	**Amino/acidic sugars**		L-Arginine	+	Sodium Bromate	-
D-Salicin	+	3-Methyl Glucose	+	L-Aspartic Acid	+	**Polyvalent alcohols**	
D-Trehalose	+	Acetic Acid	-	L-Glutamic Acid	+	D-Arabitol	+
D-Turanose	+	Acetoacetic Acid	+	L-Histidine	+	D-Mannitol	+
Dextrin	-	Bromo Succinic Acid	+	L-Pyroglutamic Acid	+	D-Sorbitol	+
Gentiobiose	-	D-Malic Acid	-	L-Serine	-	Glycerol	+
Inosine	+	Formic Acid	-	γ-Amino-Butyric Acid	-	*myo*-Inositol	+
L-Fucose	+	Glucuronamide	-	**Tetrazolium dyes**		**Others**	
L-Rhamnose	+	L-Malic Acid	-	Tetrazolium Blue	-	1% NaCl	+
Stachyose	-	Mucic Acid	+	Tetrazolium Violet	-	4% NaCl	+
Sucrose	+	N-Acetyl-D-Glucosamine	+	**Surfactants**		8% NaCl	-
α-D-Glucose	+	N-Acetylgalactosamine	+	Niaproof 4	-	Gelatin	-
α-D-Lactose	-	N-Acetyl-Neuraminic Acid	-	Tween 40	+	Guanidine HCl	-
β-Methyl-D-Glucoside	+	N-Acetyl-β-D-Mannosamine	-	**Hexose-PO_4**		pH 5	-
**Hexose acids**		Nalidixic Acid	+	D-Fructose-6-PO_4	+	pH 6	-
D-Galacturonic Acid	+	Quinic Acid	+	D-Glucose-6-PO_4	+		
D-Gluconic Acid	+	Propionic Acid	-	**Antibiotics**			
D-Glucuronic Acid	-	Sodium Butyrate	-	Aztreonam	-		

Supplementary Table S2. **Genomic insights of *Rhodoglobus galactosidasius* CPHL_1^T**. Gene sequences involved in coding for cold tolerance activities (A), glycoside hydrolases (B), and pigments (C) in the annotate genome. Locus tags from Prokka annotation. n.d., not determined.

	**Locus tag**	**Length (bp)**	**Gene**	**EC number**	**COG**	**Product**
A	00252	1647	*rhlE_1*	3.6.4.13	COG0513	ATP-dependent RNA helicase
	00257	4668	*rhlE_2*	3.6.4.13	COG0513	ATP-dependent RNA helicase
	00262	2154	*rhlE_3*	3.6.4.13	COG0513	ATP-dependent RNA helicase
	00395	564	*cshA_1*	3.6.4.13	COG0513	DEAD-box ATP-dependent RNA helicase
	00407	2160	*deadD_1*	3.6.4.13	COG0513	DEAD-box ATP-dependent RNA helicase
	00645	204	*cspA*	n.d.	COG1278	Cold shock protein A
	01590	2124	*fadB*	n.d.	n.d.	Fatty acid oxidation complex subunit α
	02219	1653	*cshA_2*	3.6.4.13	COG0513	DEAD-box ATP-dependent RNA helicase
	02847	1929	*deadD_2*	3.6.4.13	COG0513	DEAD-box ATP-dependent RNA helicase
B	00328	2040	n.d.	3.2.1.23	n.d.	β-galactosidase
	01462	1980	*malZ*	3.2.1.20	COG0366	Maltodextrin glucosidase
	01466	1788	*malL*	3.2.1.10	COG0366	Oligo-1,6-glucosidase
	01716	1797	n.d.	3.2.1.28	COG3387	Trehalase
	01718	1158	*nagZ*	3.2.1.52	n.d.	β-hexosaminidase
	02381	1857	n.d.	3.2.1.28	COG3387	Trehalase
	02596	1434	n.d.	3.2.1.21	n.d.	Bifunctional β-D-glucosidase/ β-D-fucosidase
	02945	1419	n.d.	3.2.1.21	n.d.	Bifunctional β-D-glucosidase/ β-D-fucosidase
C	01884	1584	*carA2*	1.3.99.29	COG1233	Phytoene desaturase (ζ-carotene-forming)
	02565	1062	*pks11*	2.3.1.-	COG3424	α-pyrone synthesis polyketide synthase-like
	01886	525	*idi*	5.3.3.2	COG1443	Isopentenyl-diphosphate δ-isomerase
